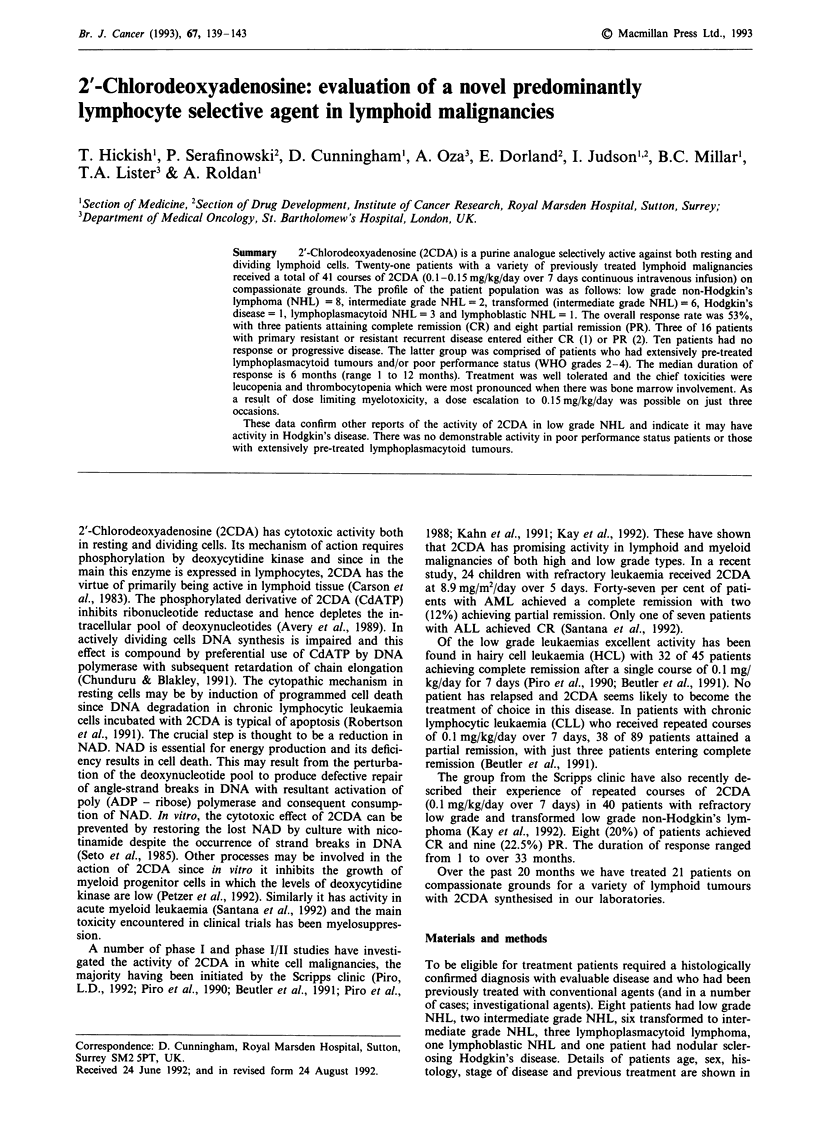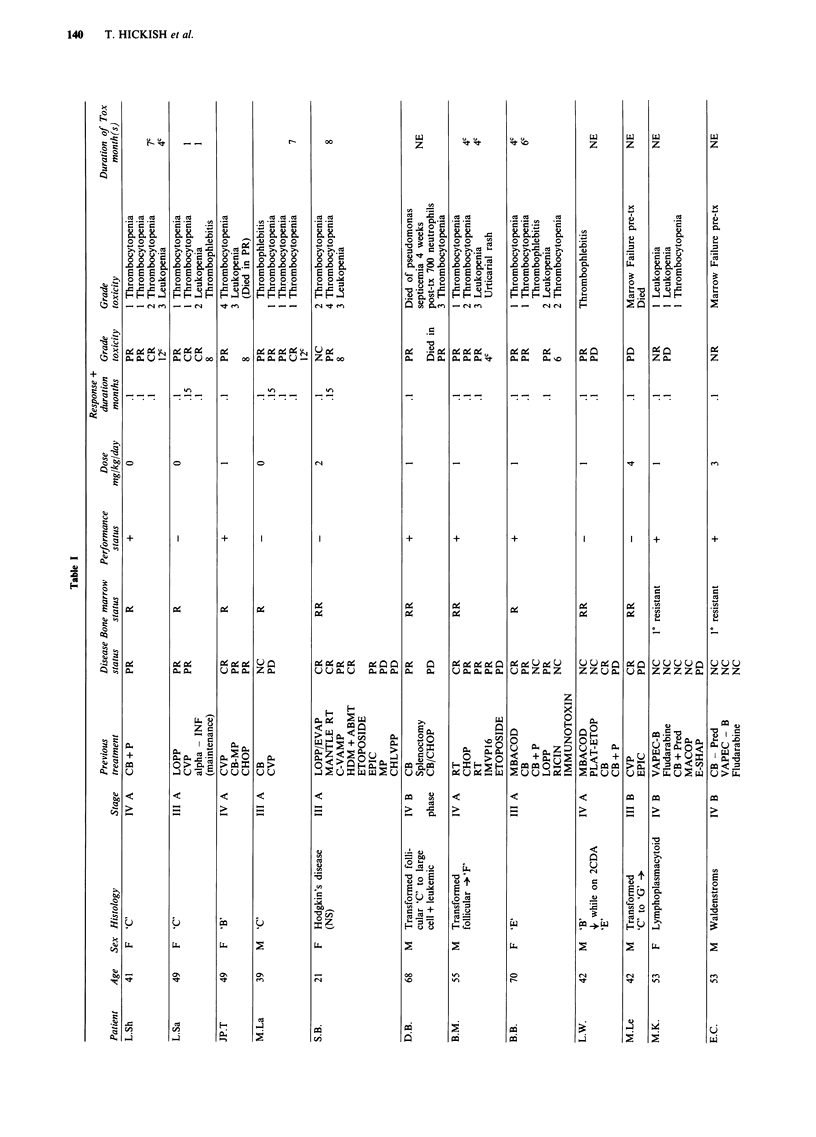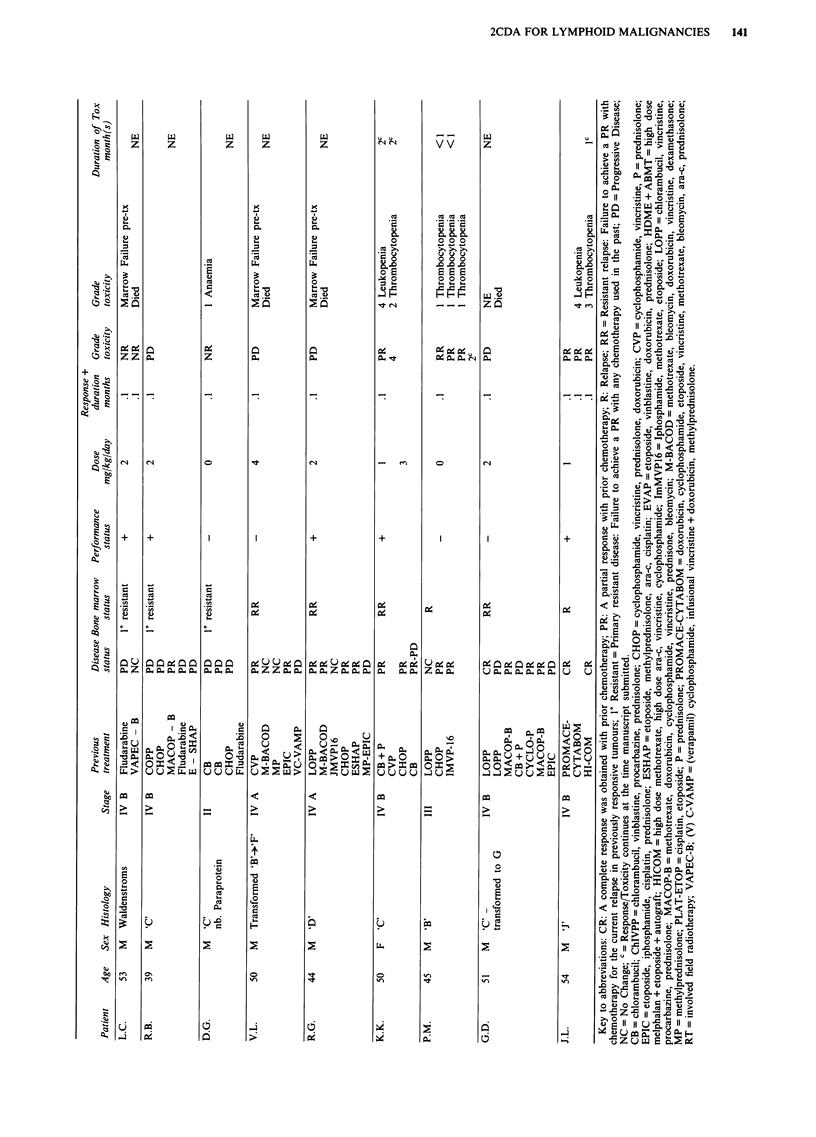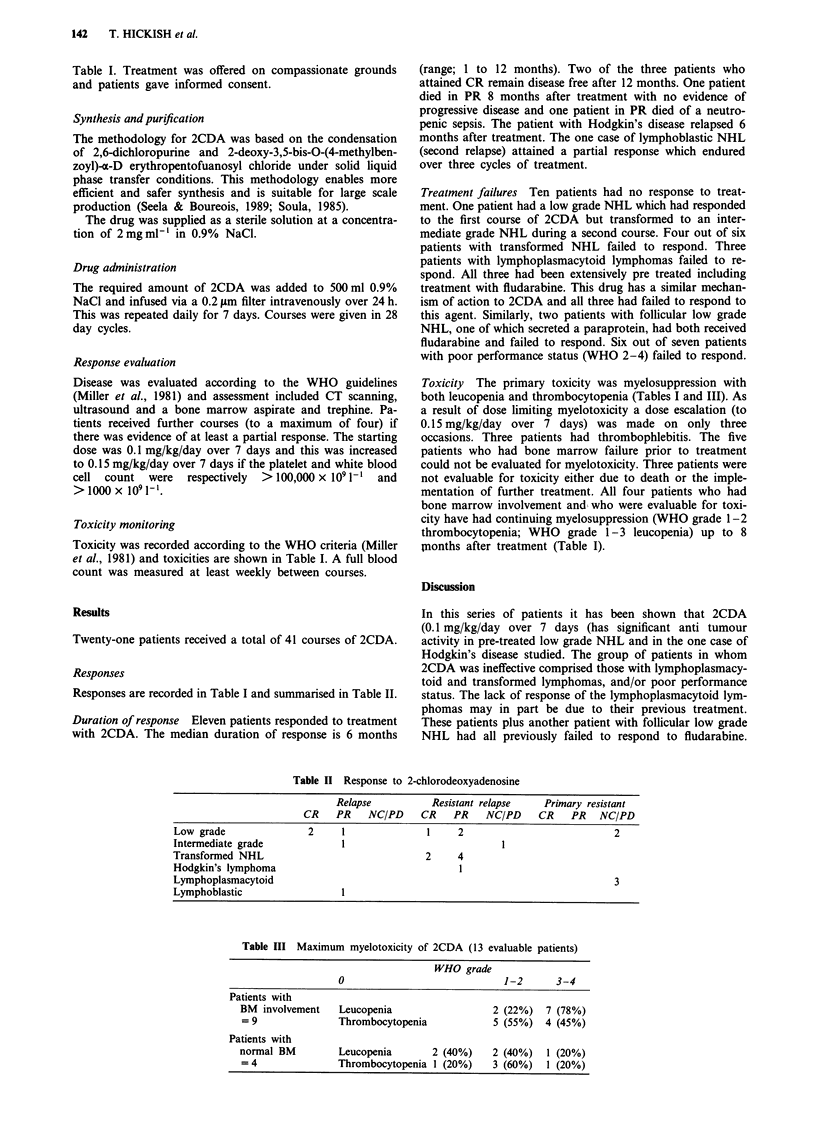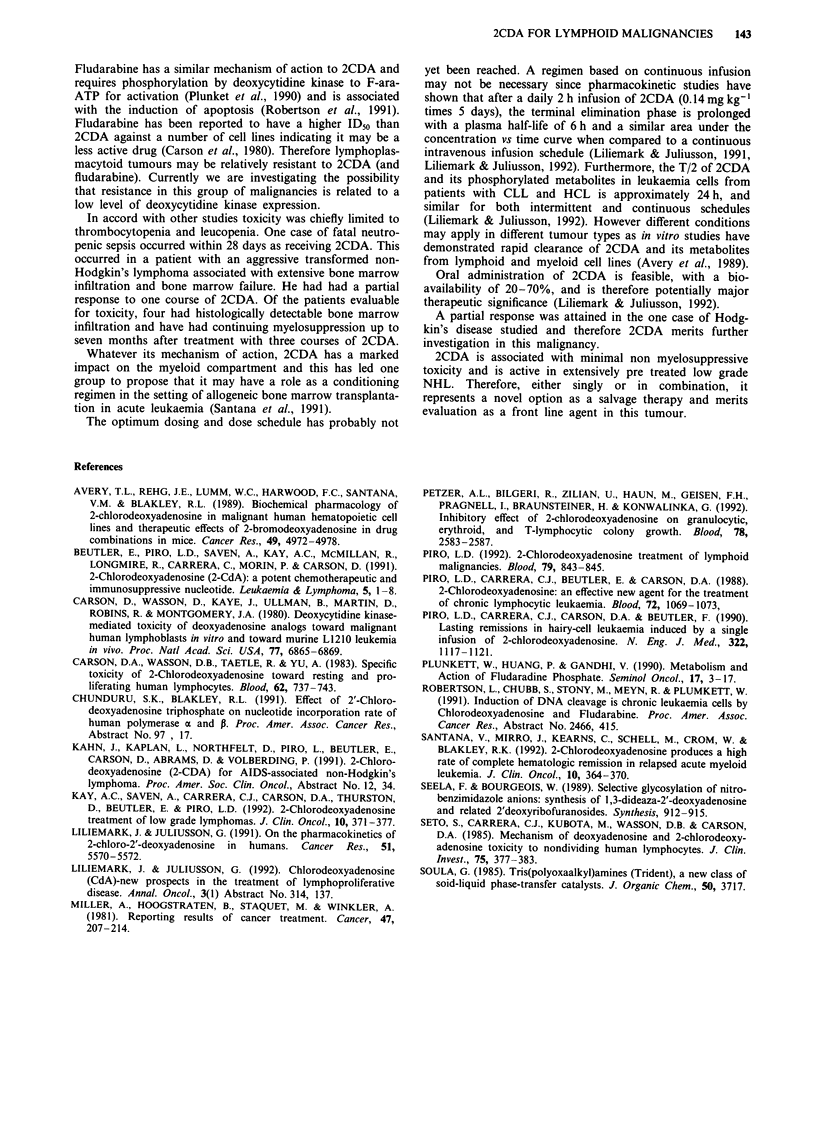# 2'-Chlorodeoxyadenosine: evaluation of a novel predominantly lymphocyte selective agent in lymphoid malignancies.

**DOI:** 10.1038/bjc.1993.24

**Published:** 1993-01

**Authors:** T. Hickish, P. Serafinowski, D. Cunningham, A. Oza, E. Dorland, I. Judson, B. C. Millar, T. A. Lister, A. Roldan

**Affiliations:** Section of Medicine, Royal Marsden Hospital, Sutton, Surrey, UK.

## Abstract

2'-Chlorodeoxyadenosine (2CDA) is a purine analogue selectively active against both resting and dividing lymphoid cells. Twenty-one patients with a variety of previously treated lymphoid malignancies received a total of 41 courses of 2CDA (0.1-0.15 mg/kg/day over 7 days continuous intravenous infusion) on compassionate grounds. The profile of the patient population was as follows: low grade non-Hodgkin's lymphoma (NHL) = 8, intermediate grade NHL = 2, transformed (intermediate grade NHL) = 6, Hodgkin's disease = 1, lymphoplasmacytoid NHL = 3 and lymphoblastic NHL = 1. The overall response rate was 53%, with three patients attaining complete remission (CR) and eight partial remission (PR). Three of 16 patients with primary resistant or resistant recurrent disease entered either CR (1) or PR (2). Ten patients had no response or progressive disease. The latter group was comprised of patients who had extensively pre-treated lymphoplasmacytoid tumours and/or poor performance status (WHO grades 2-4). The median duration of response is 6 months (range 1 to 12 months). Treatment was well tolerated and the chief toxicities were leucopenia and thrombocytopenia which were most pronounced when there was bone marrow involvement. As a result of dose limiting myelotoxicity, a dose escalation to 0.15 mg/kg/day was possible on just three occasions. These data confirm other reports of the activity of 2CDA in low grade NHL and indicate it may have activity in Hodgkin's disease. There was no demonstrable activity in poor performance status patients or those with extensively pre-treated lymphoplasmacytoid tumours.


Br. J. Cancer (1993), 67, 139–143 © Macmillan Press Ltd., 1993

### 2′-Chlorodeoxyadenosine: evaluation of a novel predominantly lymphocyte selective agent in lymphoid malignancies

T. Hickish[1], P. Serafinowski[2], D. Cunningham[1], A. Oza[3], E. Dorland[2], I. Judson[1,2], B.C. Millar[1], T.A. Lister[3] & A. Roldan[1]

[1]*Section of Medicine,* [2]*Section of Drug Development, Institute of Cancer Research, Royal Marsden Hospital, Sutton, Surrey;* [3]*Department of Medical Oncology, St. Bartholomew's Hospital, London, UK.*

**Summary** 2′-Chlorodeoxyadenosine (2CDA) is a purine analogue selectively active against both resting and dividing lymphoid cells. Twenty-one patients with a variety of previously treated lymphoid malignancies received a total of 41 courses of 2CDA (0.1–0.15 mg/kg/day over 7 days continuous intravenous infusion) on compassionate grounds. The profile of the patient population was as follows: low grade non-Hodgkin's lymphoma (NHL) = 8, intermediate grade NHL = 2, transformed (intermediate grade NHL) = 6, Hodgkin's disease = 1, lymphoplasmacytoid NHL = 3 and lymphoblastic NHL = 1. The overall response rate was 53%, with three patients attaining complete remission (CR) and eight partial remission (PR). Three of 16 patients with primary resistant or resistant recurrent disease entered either CR (1) or PR (2). Ten patients had no response or progressive disease. The latter group was comprised of patients who had extensively pre-treated lymphoplasmacytoid tumours and/or poor performance status (WHO grades 2–4). The median duration of response is 6 months (range 1 to 12 months). Treatment was well tolerated and the chief toxicities were leucopenia and thrombocytopenia which were most pronounced when there was bone marrow involvement. As a result of dose limiting myelotoxicity, a dose escalation to 0.15 mg/kg/day was possible on just three occasions.

These data confirm other reports of the activity of 2CDA in low grade NHL and indicate it may have activity in Hodgkin's disease. There was no demonstrable activity in poor performance status patients or those with extensively pre-treated lymphoplasmacytoid tumours.

2′-Chlorodeoxyadenosine (2CDA) has cytotoxic activity both in resting and dividing cells. Its mechanism of action requires phosphorylation by deoxycytidine kinase and since in the main this enzyme is expressed in lymphocytes, 2CDA has the virtue of primarily being active in lymphoid tissue (Carson *et al.*, 1983). The phosphorylated derivative of 2CDA (CdATP) inhibits ribonucleotide reductase and hence depletes the intracellular pool of deoxynucleotides (Avery *et al.*, 1989). In actively dividing cells DNA synthesis is impaired and this effect is compound by preferential use of CdATP by DNA polymerase with subsequent retardation of chain elongation (Chunduru & Blakley, 1991). The cytopathic mechanism in resting cells may be by induction of programmed cell death since DNA degradation in chronic lymphocytic leukaemia cells incubated with 2CDA is typical of apoptosis (Robertson *et al.*, 1991). The crucial step is thought to be a reduction in NAD. NAD is essential for energy production and its deficiency results in cell death. This may result from the perturbation of the deoxynucleotide pool to produce defective repair of angle-strand breaks in DNA with resultant activation of poly (ADP – ribose) polymerase and consequent consumption of NAD. *In vitro*, the cytotoxic effect of 2CDA can be prevented by restoring the lost NAD by culture with nicotinamide despite the occurrence of strand breaks in DNA (Seto *et al.*, 1985). Other processes may be involved in the action of 2CDA since *in vitro* it inhibits the growth of myeloid progenitor cells in which the levels of deoxycytidine kinase are low (Petzer *et al.*, 1992). Similarly it has activity in acute myeloid leukaemia (Santana *et al.*, 1992) and the main toxicity encountered in clinical trials has been myelosuppression.

A number of phase I and phase I/II studies have investigated the activity of 2CDA in white cell malignancies, the majority having been initiated by the Scripps clinic (Piro, L.D., 1992; Piro *et al.*, 1990; Beutler *et al.*, 1991; Piro *et al.*, 1988; Kahn *et al.*, 1991; Kay *et al.*, 1992). These have shown that 2CDA has promising activity in lymphoid and myeloid malignancies of both high and low grade types. In a recent study, 24 children with refractory leukaemia received 2CDA at 8.9 mg/m$^2$/day over 5 days. Forty-seven per cent of patients with AML achieved a complete remission with two (12%) achieving partial remission. Only one of seven patients with ALL achieved CR (Santana *et al.*, 1992).

Of the low grade leukaemias excellent activity has been found in hairy cell leukaemia (HCL) with 32 of 45 patients achieving complete remission after a single course of 0.1 mg/kg/day for 7 days (Piro *et al.*, 1990; Beutler *et al.*, 1991). No patient has relapsed and 2CDA seems likely to become the treatment of choice in this disease. In patients with chronic lymphocytic leukaemia (CLL) who received repeated courses of 0.1 mg/kg/day over 7 days, 38 of 89 patients attained a partial remission, with just three patients entering complete remission (Beutler *et al.*, 1991).

The group from the Scripps clinic have also recently described their experience of repeated courses of 2CDA (0.1 mg/kg/day over 7 days) in 40 patients with refractory low grade and transformed low grade non-Hodgkin's lymphoma (Kay *et al.*, 1992). Eight (20%) of patients achieved CR and nine (22.5%) PR. The duration of response ranged from 1 to over 33 months.

Over the past 20 months we have treated 21 patients on compassionate grounds for a variety of lymphoid tumours with 2CDA synthesised in our laboratories.

### Materials and methods

To be eligible for treatment patients required a histologically confirmed diagnosis with evaluable disease and who had been previously treated with conventional agents (and in a number of cases; investigational agents). Eight patients had low grade NHL, two intermediate grade NHL, six transformed to intermediate grade NHL, three lymphoplasmacytoid lymphoma, one lymphoblastic NHL and one patient had nodular sclerosing Hodgkin's disease. Details of patients age, sex, histology, stage of disease and previous treatment are shown in

Correspondence: D. Cunningham, Royal Marsden Hospital, Sutton, Surrey SM2 5PT, UK.
Received 24 June 1992; and in revised form 24 August 1992.

Table I. Treatment was offered on compassionate grounds and patients gave informed consent.

### Synthesis and purification

The methodology for 2CDA was based on the condensation of 2,6-dichloropurine and 2-deoxy-3,5-bis-O-(4-methylbenzoyl)-α-D erythropentofuanosyl chloride under solid liquid phase transfer conditions. This methodology enables more efficient and safer synthesis and is suitable for large scale production (Seela & Boureois, 1989; Soula, 1985).

The drug was supplied as a sterile solution at a concentration of 2 mg $ml^{-1}$ in 0.9% NaCl.

### Drug administration

The required amount of 2CDA was added to 500 ml 0.9% NaCl and infused via a 0.2 μm filter intravenously over 24 h. This was repeated daily for 7 days. Courses were given in 28 day cycles.

### Response evaluation

Disease was evaluated according to the WHO guidelines (Miller *et al.*, 1981) and assessment included CT scanning, ultrasound and a bone marrow aspirate and trephine. Patients received further courses (to a maximum of four) if there was evidence of at least a partial response. The starting dose was 0.1 mg/kg/day over 7 days and this was increased to 0.15 mg/kg/day over 7 days if the platelet and white blood cell count were respectively $> 100,000 \times 10^9 l^{-1}$ and $> 1000 \times 10^9 l^{-1}$.

### Toxicity monitoring

Toxicity was recorded according to the WHO criteria (Miller *et al.*, 1981) and toxicities are shown in Table I. A full blood count was measured at least weekly between courses.

### Results

Twenty-one patients received a total of 41 courses of 2CDA.

### Responses

Responses are recorded in Table I and summarised in Table II.

*Duration of response* Eleven patients responded to treatment with 2CDA. The median duration of response is 6 months (range; 1 to 12 months). Two of the three patients who attained CR remain disease free after 12 months. One patient died in PR 8 months after treatment with no evidence of progressive disease and one patient in PR died of a neutropenic sepsis. The patient with Hodgkin's disease relapsed 6 months after treatment. The one case of lymphoblastic NHL (second relapse) attained a partial response which endured over three cycles of treatment.

*Treatment failures* Ten patients had no response to treatment. One patient had a low grade NHL which had responded to the first course of 2CDA but transformed to an intermediate grade NHL during a second course. Four out of six patients with transformed NHL failed to respond. Three patients with lymphoplasmacytoid lymphomas failed to respond. All three had been extensively pre treated including treatment with fludarabine. This drug has a similar mechanism of action to 2CDA and all three had failed to respond to this agent. Similarly, two patients with follicular low grade NHL, one of which secreted a paraprotein, had both received fludarabine and failed to respond. Six out of seven patients with poor performance status (WHO 2–4) failed to respond.

*Toxicity* The primary toxicity was myelosuppression with both leucopenia and thrombocytopenia (Tables I and III). As a result of dose limiting myelotoxicity a dose escalation (to 0.15 mg/kg/day over 7 days) was made on only three occasions. Three patients had thrombophlebitis. The five patients who had bone marrow failure prior to treatment could not be evaluated for myelotoxicity. Three patients were not evaluable for toxicity either due to death or the implementation of further treatment. All four patients who had bone marrow involvement and who were evaluable for toxicity have had continuing myelosuppression (WHO grade 1–2 thrombocytopenia; WHO grade 1–3 leucopenia) up to 8 months after treatment (Table I).

### Discussion

In this series of patients it has been shown that 2CDA (0.1 mg/kg/day over 7 days (has significant anti tumour activity in pre-treated low grade NHL and in the one case of Hodgkin's disease studied. The group of patients in whom 2CDA was ineffective comprised those with lymphoplasmacytoid and transformed lymphomas, and/or poor performance status. The lack of response of the lymphoplasmacytoid lymphomas may in part be due to their previous treatment. These patients plus another patient with follicular low grade NHL had all previously failed to respond to fludarabine.

**Table II** Response to 2-chlorodeoxyadenosine

| | *Relapse* | | | *Resistant relapse* | | | *Primary resistant* | | |
|---|---|---|---|---|---|---|---|---|---|
| | *CR* | *PR* | *NC/PD* | *CR* | *PR* | *NC/PD* | *CR* | *PR* | *NC/PD* |
| Low grade | 2 | 1 | | 1 | 2 | | | | 2 |
| Intermediate grade | | 1 | | | | 1 | | | |
| Transformed NHL | | | | 2 | 4 | | | | |
| Hodgkin's lymphoma | | | | | 1 | | | | |
| Lymphoplasmacytoid | | | | | | | | | 3 |
| Lymphoblastic | | 1 | | | | | | | |

**Table III** Maximum myelotoxicity of 2CDA (13 evaluable patients)

| | | *WHO grade* | | |
|---|---|---|---|---|
| | | *0* | *1–2* | *3–4* |
| Patients with BM involvement = 9 | Leucopenia | | 2 (22%) | 7 (78%) |
| | Thrombocytopenia | | 5 (55%) | 4 (45%) |
| Patients with normal BM = 4 | Leucopenia | 2 (40%) | 2 (40%) | 1 (20%) |
| | Thrombocytopenia | 1 (20%) | 3 (60%) | 1 (20%) |

Fludarabine has a similar mechanism of action to 2CDA and requires phosphorylation by deoxycytidine kinase to F-ara-ATP for activation (Plunket *et al.*, 1990) and is associated with the induction of apoptosis (Robertson *et al.*, 1991). Fludarabine has been reported to have a higher $ID_{50}$ than 2CDA against a number of cell lines indicating it may be a less active drug (Carson *et al.*, 1980). Therefore lymphoplasmacytoid tumours may be relatively resistant to 2CDA (and fludarabine). Currently we are investigating the possibility that resistance in this group of malignancies is related to a low level of deoxycytidine kinase expression.

In accord with other studies toxicity was chiefly limited to thrombocytopenia and leucopenia. One case of fatal neutropenic sepsis occurred within 28 days as receiving 2CDA. This occurred in a patient with an aggressive transformed non-Hodgkin's lymphoma associated with extensive bone marrow infiltration and bone marrow failure. He had had a partial response to one course of 2CDA. Of the patients evaluable for toxicity, four had histologically detectable bone marrow infiltration and have had continuing myelosuppression up to seven months after treatment with three courses of 2CDA.

Whatever its mechanism of action, 2CDA has a marked impact on the myeloid compartment and this has led one group to propose that it may have a role as a conditioning regimen in the setting of allogeneic bone marrow transplantation in acute leukaemia (Santana *et al.*, 1991).

The optimum dosing and dose schedule has probably not yet been reached. A regimen based on continuous infusion may not be necessary since pharmacokinetic studies have shown that after a daily 2 h infusion of 2CDA (0.14 mg $kg^{-1}$ times 5 days), the terminal elimination phase is prolonged with a plasma half-life of 6 h and a similar area under the concentration *vs* time curve when compared to a continuous intravenous infusion schedule (Liliemark & Juliusson, 1991, Liliemark & Juliusson, 1992). Furthermore, the T/2 of 2CDA and its phosphorylated metabolites in leukaemia cells from patients with CLL and HCL is approximately 24 h, and similar for both intermittent and continuous schedules (Liliemark & Juliusson, 1992). However different conditions may apply in different tumour types as *in vitro* studies have demonstrated rapid clearance of 2CDA and its metabolites from lymphoid and myeloid cell lines (Avery *et al.*, 1989).

Oral administration of 2CDA is feasible, with a bioavailability of 20–70%, and is therefore potentially major therapeutic significance (Liliemark & Juliusson, 1992).

A partial response was attained in the one case of Hodgkin's disease studied and therefore 2CDA merits further investigation in this malignancy.

2CDA is associated with minimal non myelosuppressive toxicity and is active in extensively pre treated low grade NHL. Therefore, either singly or in combination, it represents a novel option as a salvage therapy and merits evaluation as a front line agent in this tumour.

### References

AVERY, T.L., REHG, J.E., LUMM, W.C., HARWOOD, F.C., SANTANA, V.M. & BLAKLEY, R.L. (1989). Biochemical pharmacology of 2-chlorodeoxyadenosine in malignant human hematopoietic cell lines and therapeutic effects of 2-bromodeoxyadenosine in drug combinations in mice. *Cancer Res.*, **49**, 4972–4978.

BEUTLER, E., PIRO, L.D., SAVEN, A., KAY, A.C., MCMILLAN, R., LONGMIRE, R., CARRERA, C., MORIN, P. & CARSON, D. (1991). 2-Chlorodeoxyadenosine (2-CdA): a potent chemotherapeutic and immunosuppressive nucleotide. *Leukaemia & Lymphoma*, **5**, 1–8.

CARSON, D., WASSON, D., KAYE, J., ULLMAN, B., MARTIN, D., ROBINS, R. & MONTGOMERY, J.A. (1980). Deoxycytidine kinase-mediated toxicity of deoxyadenosine analogs toward malignant human lymphoblasts *in vitro* and toward murine L1210 leukemia *in vivo*. *Proc. Natl Acad. Sci. USA*, **77**, 6865–6869.

CARSON, D.A., WASSON, D.B., TAETLE, R. & YU, A. (1983). Specific toxicity of 2-Chlorodeoxyadenosine toward resting and proliferating human lymphocytes. *Blood*, **62**, 737–743.

CHUNDURU, S.K., BLAKLEY, R.L. (1991). Effect of 2′-Chlorodeoxyadenosine triphosphate on nucleotide incorporation rate of human polymerase α and β. *Proc. Amer. Assoc. Cancer Res.*, Abstract No. 97 , 17.

KAHN, J., KAPLAN, L., NORTHFELT, D., PIRO, L., BEUTLER, E., CARSON, D., ABRAMS, D. & VOLBERDING, P. (1991). 2-Chlorodeoxyadenosine (2-CDA) for AIDS-associated non-Hodgkin's lymphoma. *Proc. Amer. Soc. Clin. Oncol.*, Abstract No. 12, 34.

KAY, A.C., SAVEN, A., CARRERA, C.J., CARSON, D.A., THURSTON, D., BEUTLER, E. & PIRO, L.D. (1992). 2-Chlorodeoxyadenosine treatment of low grade lymphomas. *J. Clin. Oncol.*, **10**, 371–377.

LILIEMARK, J. & JULIUSSON, G. (1991). On the pharmacokinetics of 2-chloro-2′-deoxyadenosine in humans. *Cancer Res.*, **51**, 5570–5572.

LILIEMARK, J. & JULIUSSON, G. (1992). Chlorodeoxyadenosine (CdA)-new prospects in the treatment of lymphoproliferative disease. *Annal. Oncol.*, **3**(1) Abstract No. 314, 137.

MILLER, A., HOOGSTRATEN, B., STAQUET, M. & WINKLER, A. (1981). Reporting results of cancer treatment. *Cancer*, **47**, 207–214.

PETZER, A.L., BILGERI, R., ZILIAN, U., HAUN, M., GEISEN, F.H., PRAGNELL, I., BRAUNSTEINER, H. & KONWALINKA, G. (1992). Inhibitory effect of 2-chlorodeoxyadenosine on granulocytic, erythroid, and T-lymphocytic colony growth. *Blood*, **78**, 2583–2587.

PIRO, L.D. (1992). 2-Chlorodeoxyadenosine treatment of lymphoid malignancies. *Blood*, **79**, 843–845.

PIRO, L.D., CARRERA, C.J., BEUTLER, E. & CARSON, D.A. (1988). 2-Chlorodeoxyadenosine: an effective new agent for the treatment of chronic lymphocytic leukaemia. *Blood*, **72**, 1069–1073,

PIRO, L.D., CARRERA, C.J., CARSON, D.A. & BEUTLER, F. (1990). Lasting remissions in hairy-cell leukaemia induced by a single infusion of 2-chlorodeoxyadenosine. *N. Eng. J. Med.*, **322**, 1117–1121.

PLUNKETT, W., HUANG, P. & GANDHI, V. (1990). Metabolism and Action of Fludaradine Phosphate. *Seminol Oncol.*, **17**, 3–17.

ROBERTSON, L., CHUBB, S., STONY, M., MEYN, R. & PLUMKETT, W. (1991). Induction of DNA cleavage is chronic leukaemia cells by Chlorodeoxyadenosine and Fludarabine. *Proc. Amer. Assoc. Cancer Res.*, Abstract No. 2466, 415.

SANTANA, V., MIRRO, J., KEARNS, C., SCHELL, M., CROM, W. & BLAKLEY, R.K. (1992). 2-Chlorodeoxyadenosine produces a high rate of complete hematologic remission in relapsed acute myeloid leukemia. *J. Clin. Oncol.*, **10**, 364–370.

SEELA, F. & BOURGEOIS, W. (1989). Selective glycosylation of nitrobenzimidazole anions: synthesis of 1,3-dideaza-2′-deoxyadenosine and related 2′deoxyribofuranosides. *Synthesis*, 912–915.

SETO, S., CARRERA, C.J., KUBOTA, M., WASSON, D.B. & CARSON, D.A. (1985). Mechanism of deoxyadenosine and 2-chlorodeoxyadenosine toxicity to nondividing human lymphocytes. *J. Clin. Invest.*, **75**, 377–383.

SOULA, G. (1985). Tris(polyoxaalkyl)amines (Trident), a new class of soid-liquid phase-transfer catalysts. *J. Organic Chem.*, **50**, 3717.